# Alternative use of multiple exons 1 of aromatase gene in cancerous and normal breast tissues from women over the age of 80 years

**DOI:** 10.1186/bcr2335

**Published:** 2009-07-10

**Authors:** Naoko Honma, Kaiyo Takubo, Motoji Sawabe, Tomio Arai, Futoshi Akiyama, Goi Sakamoto, Toshiaki Utsumi, Noriko Yoshimura, Nobuhiro Harada

**Affiliations:** 1 Research Team for Geriatric Diseases, Tokyo Metropolitan Institute of Gerontology, Sakaecho 35-2, Itabashi-ku, Tokyo 173-0015, Japan; 2 Department of Pathology, Tokyo Metropolitan Geriatric Medical Center, Sakaecho 35-2, Itabashi-ku, Tokyo 173-0015, Japan; 3 Department of Breast Pathology, Cancer Institute, Ariake 3-10-6, Koto-ku, Tokyo 135-8550, Japan; 4 Department of Surgery, Fujita Health University School of Medicine, Dengakugakubo 1-98, Kutsukake-cho, Toyoake, Aichi 470-1192, Japan; 5 Department of Biochemistry, Fujita Health University School of Medicine, Dengakugakubo 1-98, Kutsukake-cho, Toyoake, Aichi 470-1192, Japan

## Abstract

**Introduction:**

Peripherally localized aromatase, which converts circulating androgens into estrogens, is important in the pathogenesis of postmenopausal breast carcinomas. We have previously shown that aromatase mRNA levels are higher in elderly breast carcinomas (EldCa) than breast carcinomas of the control group (ContCa) or normal breast tissues. Aromatase expression has been reported to be regulated through the alternative use of multiple exons 1 (exons 1a-1f and so on); however, the preferential usage of exons 1 in elderly breast tissue has never been systematically examined. In order to properly treat and protect against EldCa, the regulation mechanism of aromatase expression in elderly breast tissues should be elucidated. The aim of the present study is to elucidate whether there are any specific patterns in use of multiple exons 1 in elderly breast tissue.

**Methods:**

Usage of multiple exons 1 of the aromatase gene and mRNA levels of aromatase were examined by reverse transcription-polymerase chain reaction analysis in breast tissues of 38 elderly patients with breast cancer (age 80–99), and the results were compared with those in 35 patients of the control group (age 37–70). One-factor analysis of variance and the Scheffé test were used for the comparison of aromatase mRNA levels. Patterns of preferential utilization of multiple exons 1 of the aromatase gene were compared by χ^2 ^test for independence or Fisher exact test for independence using a contingency table.

**Results:**

Exon 1d was utilized much more frequently in elderly tissue than in the control group irrespective of cancerous or normal tissue (EldCa, 36/38, 95% versus ContCa, 7/35, 20%, *P *< 0.0001; normal tissue of the elderly, EldNorm, 30/34, 88% versus normal tissue of controls, ContNorm, 2/29, 7%, *P *< 0.0001). Twenty EldCa (53%) and 12 EldNorm (35%) used both exons 1c and 1d; however, their dominance was reversed (EldCa, all 1d > 1c; EldNorm, all 1c > 1d).

**Conclusions:**

Elderly breast tissues exhibited specific patterns in use of multiple exons 1, which at least partly explained the higher aromatase levels in EldCa. The mechanisms of how these specific patterns occur during aging and carcinogenesis should be further examined.

## Introduction

Estrogen plays important roles in the pathogenesis and development of breast cancer [1]. In postmenopausal women, in whom ovarian function has decreased, the peripheral metabolism and biosynthesis of estrogens via estrogen-metabolizing enzymes are important. Among several estrogen-metabolizing enzymes involved in the pathogenesis of breast cancer [2-10], aromatase is a key enzyme, converting circulating androgens from the adrenal gland or ovary into estrogens [2]. The application of aromatase inhibitors is already a standardized treatment for postmenopausal breast carcinomas [11-14].

Physiologically, aromatase exists not only in gonadal tissues such as the ovary and placenta, but also in extragonadal tissues such as the brain [15], skin fibroblasts [16], and adipose tissue [17]. The tissue-specific expression of aromatase is strictly regulated; however, it is difficult to explain the complex transcriptional regulation of the aromatase gene in various tissues by a single gene and a single promoter. Multiple tissue-specific promoters of the aromatase gene were first shown by Means and colleagues [18] and Mahendroo and colleagues [19]. We reported that the aromatase gene was regulated tissue-specifically through the alternative use of multiple exons 1 [20]. Exons 1a (I.1), 1b (I.4), 1c (I.3), and 1d (P.II) are used mainly for aromatase mRNAs of the placenta, skin fibroblast/fetal liver, ovary, and prostate/ovary, respectively. Each of the multiple exons 1 of the human aromatase gene is flanked by a unique promoter region [21]. In regard to breast carcinogenesis, switching of the preferential utilization of multiple exons 1, exon 1b to exon 1c/1d, has been suggested to be responsible for the abnormal expression of aromatase and consequent overproduction of local estrogen in tumors [22,23].

Breast carcinoma in very elderly women is of interest because ovarian estrogens are markedly low throughout the disease process. A previous study revealed that breast carcinoma in very elderly women exhibits a distinct histologic pattern and hormone receptor status [24]. We have examined mRNA levels of several estrogen-metabolizing enzymes and have shown that levels of aromatase mRNA were significantly higher in breast carcinomas from women over the age of 80 years than in carcinomas from younger women or in normal breast tissues [25]. These findings suggest that breast carcinomas in very elderly women are quite different from those in younger women in terms of aromatase expression. As aromatase expression is regulated by multiple exons 1 of the aromatase gene, this should be elucidated in order to provide proper treatment or protect against breast carcinomas in very elderly women. In the present study, we investigate the preferential utilization of multiple exons 1 of the aromatase gene, which has never been systematically examined in breast carcinomas or normal tissues of the elderly, and compare it with that in breast tissues from younger women.

## Materials and methods

### Patients

Materials for this study were obtained from 38 Japanese patients over the age of 80 (range 80 to 99 and mean 86) years and 35 Japanese patients ages 37 to 70 (mean 53) years with primary breast carcinomas who underwent curative surgery at the Tokyo Metropolitan Geriatric Medical Center and Fujita Health University Hospital, respectively, between 1999 and 2003. All tumors were histologically classified by pathologists (NHo, KT, and GS) according to the World Health Organization classification [26]. Of the 38 carcinomas of the elderly, 24, 7, 5, and 2 were classified as invasive ductal carcinoma, apocrine carcinoma, mucinous carcinoma, and invasive lobular carcinoma, respectively, whereas all 35 carcinomas of the younger group were classified as invasive ductal carcinoma. Immediately after surgical removal, the specimens of both tumor tissues and normal tissues were frozen in liquid nitrogen and then stored at -80°C prior to use. Normal breast tissues were available in 34 elderly and 29 control cases. Materials were classified into four tissue categories: carcinomas from the elderly (EldCa), normal tissues from the elderly (EldNorm), carcinomas from controls (ContCa), and normal tissues from controls (ContNorm). Informed consent was obtained, and the study protocol was approved by the Tokyo Metropolitan Institute of Gerontology Ethics Committee.

### Quantification of aromatase mRNA

Frozen tissues were homogenized as previously reported [7], and total RNA fractions were prepared from homogenates as described by Chirgwin and colleagues [27]. Quantitative analysis of aromatase mRNA in RNA fractions was carried out by reverse transcription-polymerase chain reaction (RT-PCR) using a fluorescent primer in the presence of an internal standard RNA, as previously described [28,29]. Oligonucleotides of antisense primer 6a (5'-AACCACGATAGCACTTTCGT-3' specific for exon 6) for RT and antisense (5'-TGTTAGAGGTGTCCAGCATG-3' specific for exon 6) and sense (5'-TACTACAACCGGGTATATGG-3' specific for exon 3) primers 6b and 3a for PCR were synthesized. The sense primer 3a for PCR was labeled with a fluorescent dye, 6-carboxyfluorescein (PerkinElmer, Waltham, MA, USA). To prepare the internal standard RNA, modified human aromatase cDNA was constructed by inserting a 21-base pair DNA fragment between the two PCR primer sites. The internal standard RNA was synthesized *in vitro *with T7 RNA polymerase using modified aromatase cDNA as a template. Total RNA mixed with a known amount of internal standard RNA was subjected to RT with SuperScript II RNase H^- ^Reverse Transcriptase (Invitrogen Corporation, Carlsbad, CA, USA) and specific antisense primer 6a at 42°C for 40 minutes. The resulting cDNAs were amplified by PCR using fluorescence-labeled primers 3a and 6b. The fluorescent PCR products were electrophoresed in a 2% agarose gel and analyzed with an ABI PRISM 377 DNA Sequencer (Applied Biosystems, Foster City, CA, USA). The amount of aromatase mRNA was calculated from the peak areas of fluorescent products by the internal standard method.

### Analysis of alternative usage of exons 1 in aromatase mRNA

The utilization of multiple exons 1 of the aromatase gene in breast tissues was investigated by RT-PCR as previously described [28]. Oligonucleotide primers 1a (5'-CTGGAGGGCTGAACACGTGG-3'), specific for placenta-type exon 1 (exon 1a); 1b (5'-GACCAACTGGAGCCTGACAG-3'), specific for skin fibroblast/fetal liver-type exon 1 (exon 1b); 1c (5'-CCTTGTTTTGACTTGTAACCA-3'), specific for ovary-type exon 1 (exon 1c); and 1d (5'-AACAGGAGCTATAGATGAAC-3'), specific for ovary/prostate-type exon 1 (exon 1d), and antisense primer 3b (5'-CAGAGATCCAGACTCGCATG-3'), specific for exon 3, were synthesized.

RNA fractions from breast tissues were reverse-transcribed with SuperScript II RNase H^- ^Reverse Transcriptase and specific antisense primer 6a. The resulting cDNAs were amplified by PCR using fluorescent dye (6-carboxyfluorescein)-labeled primer 3b and primer 1a, 1b, 1c, or 1d. Fluorescent PCR products were separated by electrophoresis on a 2% agarose gel and then analyzed with an ABI PRISM 377 DNA Sequencer.

### Immunohistochemistry

Representative slides of primary carcinomas were selected for immunohistochemical examination. Antigen retrieval was performed by boiling the sections for 2 minutes in 10 mM citrate buffer (pH 6) using a pressure cooker. After the blocking of nonspecific activity, the sections were incubated with prediluted anti-estrogen receptor (anti-ER) mouse monoclonal antibody (clone 1D5; Dako, Carpinteria, CA, USA). Incubation with the anti-ER antibody was for 60 minutes at room temperature. Dako ChemMate Envision (Dako) was used for all immunohistochemical stains. A positive control slide, whose immunoreactivity had been confirmed, and a negative control slide, to which subclass-matched control IgGs instead of the specific monoclonal antibody were applied, were included in each batch. The results of immunologic staining were assessed by two pathologists (NHo and KT) independently, and where discrepancies occurred, the cases were discussed. Immunoreactivity for ER was scored by evaluating the percentage of positively stained cancer cells, irrespective of intensity; nuclear immunoreactivity in 10% or more of the cancer cells was considered positive according to St. Gallen Consensus 2003 [30].

### Statistics

Statistical analyses were carried out using StatView 5.0 (SAS Institute Inc., Cary, NC, USA). In regard to aromatase mRNA levels, one-factor analysis of variance (ANOVA) was used for the comparison of multiple groups, and the Scheffé test was used for comparisons between two of the multiple groups. For comparison of aromatase mRNA levels between mucinous carcinomas and carcinomas of other histologic types, the Student *t *test was used. Patterns of preferential utilization of multiple exons 1 of the aromatase gene (or distribution of 'Ex 1 group' as described below) were compared by chi-square test for independence or Fisher exact test for independence using a contingency table as follows: EldCa versus EldNorm, ContCa versus ContNorm, EldCa versus ContCa, and EldNorm versus ContNorm. A *P *value of less than 0.05 was considered significant.

## Results

### Comparison of aromatase mRNA levels among each tissue category

**Figure 1:**
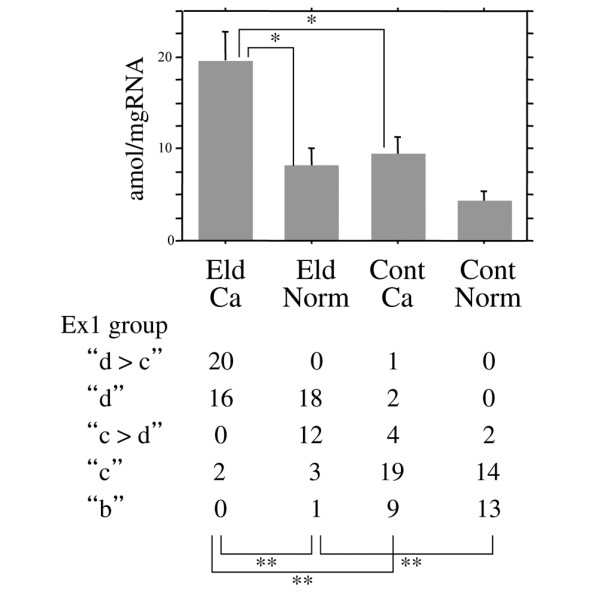
Comparison of aromatase mRNA levels and distribution of Ex 1 groups among tissue categories. Aromatase mRNA levels (top, Scheffé test) and distribution of Ex 1 groups (bottom, chi-square test for independence) were compared among carcinomas from the elderly (EldCa), normal tissues from the elderly (EldNorm), carcinomas from controls (ContCa), and normal tissues from controls (ContNorm). Bar indicates standard error of the mean. *P < 0.01; **P < 0.0001.

Levels of the mean ± standard error of mean of aromatase mRNA obtained for EldCa, EldNorm, ContCa, and ContNorm were 19.7 ± 2.97 amol/mgRNA, 8.22 ± 1.91 amol/mgRNA, 9.45 ± 1.75 amol/mgRNA, and 4.26 ± 1.09 amol/mgRNA, respectively (Table 1 and Figure 1). Aromatase mRNA levels were significantly different among the four tissue categories (one-factor ANOVA, *P *< 0.0001). When cancerous and normal tissues were compared, EldCa exhibited significantly higher aromatase levels than did EldNorm (Scheffé test, *P *= 0.0028), whereas there was no significant difference between ContCa and ContNorm (*P *= 0.4477). When the elderly and controls were compared, EldCa exhibited significantly higher aromatase levels than did ContCa (*P *= 0.0095), whereas there was no significant difference between EldNorm and ContNorm (*P *= 0.6760) (Figure 1).

**Table 1:** Aromatase mRNA levels and preferential utilization of multiple exons 1 of the aromatase gene

Exon 1 group	Tissue category								
	
	EldCa		EldNorm		ContCa		ContNorm		*P *value^a^
	
	n (%)	Aromatase mRNA ± SEM^b^	n (%)	Aromatase mRNA ± SEM^b^	n (%)	Aromatase mRNA ± SEM^b^	n (%)	Aromatase mRNA ± SEM^b^	
'd > c'	20 (53%)	26.5 ± 4.49			1 (3%)	22.8			0.8610 NS
dc	5 (13%)	35.5 ± 14.4							
dcb	1 (3%)	18.8							
dbc	7 (18%)	21.8 ± 5.79							
bdc	7 (18%)	25.8 ± 5.98			1 (3%)	22.8			

'd'	16 (42%)	12.0 ± 3.28	18 (53%)	4.67 ± 1.13	2 (6%)	10.9 ± 7.75			0.0941 NS
d	12 (32%)	13.7 ± 4.21	15 (44%)	4.13 ± 1.30					
db	2 (5%)	6.05 ± 0.350	1 (3%)	9.20					
bd	2 (5%)	8.25 ± 7.15	2 (6%)	6.45 ± 0.950	2 (6%)	10.9 ± 7.75			

'c > d'			12 (35%)	15.3 ± 4.54	4 (11%)	8.56 ± 4.57	2 (7%)	2.98 ± 1.67	0.4445 NS
cd			1 (3%)	10.5	3 (9%)	4.44 ± 2.80	2 (7%)	2.98 ± 1.67	
cdb			3 (9%)	11.2 ± 9.32					
cbd			5 (15%)	20.5 ± 8.85	1 (3%)	20.9			
bcd			3 (9%)	12.6 ± 8.18					

'c'	2 (5%)	13.1 ± 11.6	3 (9%)	3.37 ± 2.56	19 (54%)	10.8 ± 2.82	14 (48%)	7.26 ± 1.96	0.5534 NS
c	2 (5%)	13.1 ± 11.6	3 (9%)	3.37 ± 2.56	10 (29%)	9.07 ± 4.34	5 (17%)	2.04 ± 0.645	
cb					4 (11%)	17.2 ± 7.01	5 (17%)	9.33 ± 3.54	
bc					5 (14%)	9.12 ± 3.30	4 (14%)	11.2 ± 4.26	

'b' = b			1 (3%)	1.10	9 (26%)	5.21 ± 1.54	13 (45%)	1.22 ± 0.339	0.0222 S

Total	38 (100%)	19.7 ± 2.97	34 (100%)	8.22 ± 1.91	35 (100%)	9.45 ± 1.75	29 (100%)	4.26 ± 1.09	< 0.0001 S
*P *value^c^	0.0511 NS		0.0444 S		0.4871 NS		0.0209 S		

Comparison of aromatase mRNA levels according to preferential utilization of multiple exons 1 of the aromatase gene in carcinomas from the elderly (EldCa), normal tissues from the elderly (EldNorm), carcinomas from controls (ContCa), and normal tissues from controls (ContNorm). For example, dcb means exon 1d > 1c > 1b. ^a^Comparison of aromatase mRNA levels according to tissue categories in each exon 1 group or in total (one-factor analysis of variance). ^b^Values are presented in amol/mgRNA. ^c^Comparison of aromatase mRNA levels according to exon 1 groups in each tissue category (one-factor analysis of variance). NS, not significant; S, significant; SEM, standard error of the mean.

### Comparison of preferential utilization of multiple exons 1 of the aromatase gene and aromatase mRNA levels among EldCa, EldNorm, ContCa, and ContNorm

**Figure 2:**
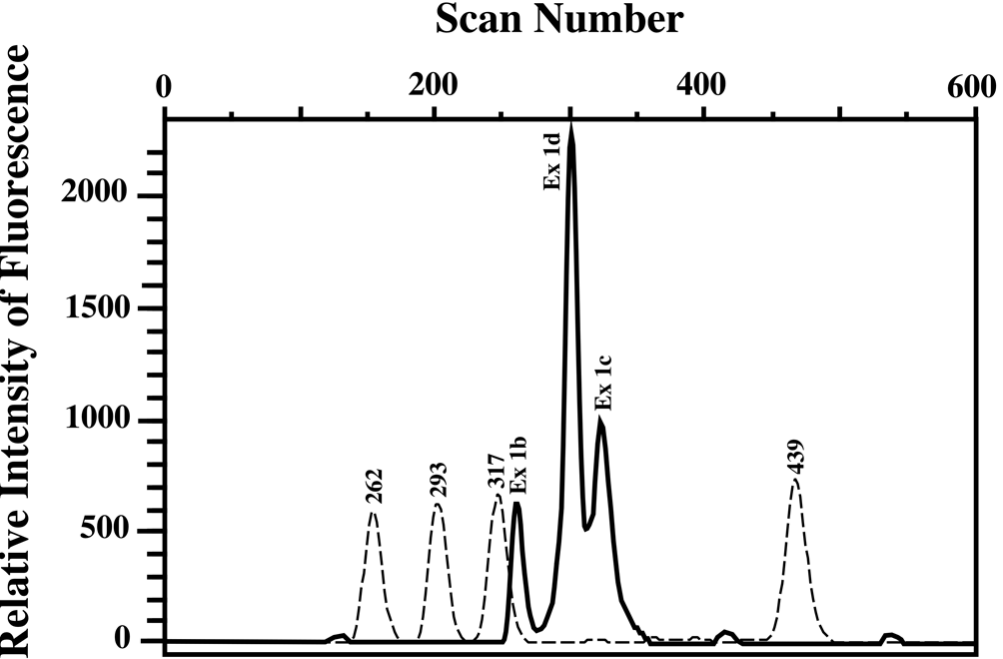
Reverse transcription-polymerase chain reaction analysis of usage of alternative exons 1 of the aromatase gene. An example of a breast cancer from elderly patients (EldCa) is shown. Exon 1d is preferentially used in the EldCa case, followed by exon 1c and exon 1b (1d > 1c > 1b, dcb, unbroken line). Broken line indicates size standards.

To determine which types of multiple exons 1 are used, RT-PCR analysis was carried out, and the relative utilization preference of exons 1 was fluorometrically determined as shown in Figure 2. Utilization of multiple exons 1 in each tissue category (EldCa, EldNorm, ContCa, and ContNorm) and mean levels of aromatase mRNA according to which exons 1 were used are summarized in Table 1. In the first column of Table 1, for example, exon 1d > 1b > 1c is conventionally designated as dbc. The proportion of cases utilizing exon 1c and/or 1d was higher in ContCa (26/35 = 74%) than in ContNorm (16/29 = 55%), although the difference was not significant (chi-square test for independence, *P *= 0.1090). Most of both EldCa (36/38 = 95%) and EldNorm (30/34 = 88%) utilized exon 1d, whereas a significantly lower proportion of ContCa (7/35 = 20%) and ContNorm (2/29 = 7%) utilized exon 1d (Fisher exact test for independence, EldCa versus ContCa, *P *< 0.0001; EldNorm versus ContNorm, *P *< 0.0001). In both EldCa and EldNorm, cases that utilized both exon 1c and exon 1d exhibited higher aromatase mRNA levels than those utilizing either exon 1c or exon 1d. Furthermore, among EldCa and EldNorm utilizing both exon 1c and exon 1d, all of EldCa exhibited exon 1d > exon 1c, whereas EldNorm exhibited the opposite result. Given the characteristic pattern of exon 1 utilization among each tissue category and aromatase mRNA levels, cases were categorized into five groups according to which exons 1 were utilized (Ex 1 groups): group 'b', which exclusively utilized exon 1b; group 'c', which utilized exon 1c but not exon 1d; group 'c > d', which utilized both exon 1c and exon 1d with dominance of exon 1c; group 'd', which utilized exon 1d but not exon 1c; and group 'd > c', which utilized both exon 1c and exon 1d with dominance of exon 1d. The presence or dominance of exon 1b did not affect aromatase mRNA levels when co-utilized with other exons 1, and grouping was regardless of exon 1b; for example, cases showing exon 1d > 1c, exon 1d > 1c > 1b, exon 1d > 1b > 1c, and exon 1b > 1d > 1c were all categorized into group 'd > c' (Table 1). The distribution of Ex 1 groups was significantly different between EldCa and EldNorm, between EldCa and ContCa, and between EldNorm and ContNorm (all *P *< 0.0001, chi-square test for independence), whereas there was no significant difference between ContCa and ContNorm (*P *= 0.3274) (Figure 1).

### Comparison of aromatase mRNA levels according to Ex 1 groups in each tissue category or according to tissue categories in each Ex 1 group

Significant or almost significant differences in aromatase mRNA levels according to Ex 1 groups were observed in EldCa (*P *= 0.0511), EldNorm (*P *= 0.0444), and ContNorm (*P *= 0.0209), whereas no significant difference was obtained in ContCa (*P *= 0.4871, one-factor ANOVA) (Table 1). There was no significant difference of aromatase mRNA levels according to tissue categories in each Ex 1 group except group 'b' (group 'd > c', *P *= 0.8610; group 'd', *P *= 0.0941; group 'c > d', *P *= 0.4445; group 'c', *P *= 0.5534; group 'b', *P *= 0.0222; one-factor ANOVA) (Table 1).

### Comparison of estrogen receptor status, aromatase mRNA levels, and distribution of Ex 1 groups according to histologic types among EldCa

Mean levels of aromatase mRNA were compared according to histologic types among EldCa (Table 2). Significant difference of aromatase levels was observed among the four histologic types (*P *= 0.0403, one-factor ANOVA). Mucinous carcinomas exhibited significantly higher levels than the other carcinomas (39.1 ± 8.99 amol/mgRNA versus 16.8 ± 2.86 amol/mgRNA, *P *= 0.0090, Student *t *test). All five mucinous carcinomas were ER-positive (Table 2). In regard to the distribution of Ex 1 groups in each histologic type (Table 2), all five mucinous carcinomas were classified as group 'd > c', showing a tendency to differ from the distribution of the other carcinomas (*P *= 0.0750, chi-square test for independence).

**Table 2:** Estrogen receptor status, aromatase mRNA levels, and exon 1 group of cancer from the elderly according to histologic types

Histologic type (n)	Estrogen receptor		Aromatase mRNA ± SEM, amol/mgRNA	Exon 1 group				
			
	+ (%)	-		d > c	d	c < d	c	b
Muc (5)	5 (100%)	0	39.1 ± 8.99	5	0	0	0	0
Others (33)	22 (67%)	11	16.8 ± 2.86	15	16	0	2	0
Apo (7)	0 (0%)	7	20.1 ± 4.06	3	3	0	1	0
ILC (2)	1 (50%)	1	2.45 ± 0.05	1	1	0	0	0
IDC (24)	21 (88%)	3	17.0 ± 3.68	11	12	0	1	0
Total (38)	27 (71%)	11	19.7 ± 2.97	20	16	0	2	0
*P *value Muc vs. others			0.0090 S	0.0750 NS				

+, positive; -, negative; Apo, apocrine carcinoma; IDC, invasive ductal carcinoma; ILC, invasive lobular carcinoma; Muc, mucinous carcinoma; NS, not significant; S, significant; SEM, standard error of the mean.

## Discussion

EldCa, EldNorm, ContCa, and ContNorm each exhibited characteristic features in the usage of multiple exons 1 of the aromatase gene. The distribution of Ex 1 groups was significantly different between EldCa and EldNorm, between EldCa and ContCa, and between EldNorm and ContNorm (all *P *< 0.0001). Mean levels of aromatase mRNA were significantly different among the four tissue categories (*P *< 0.0001), producing significant differences between EldCa and EldNorm and between EldCa and ContCa. Aromatase mRNA levels significantly or almost significantly differed according to Ex 1 groups in each tissue category except ContCa, whereas no significant difference of aromatase mRNA levels was observed according to tissue categories in any Ex 1 group except group 'b'. Those results suggest that differences of aromatase mRNA levels between tissue categories were related to the preferential usage of multiple exons 1 in each tissue category (Figure 1).

Given that exon 1d was used in a significantly larger proportion of elderly tissues than the tissues of the control group regardless of whether the tissue was cancerous or normal, the utilization of exon 1d seems to be characteristic of elderly tissue. Contrary to the control group, where exon 1b is preferentially utilized under physiologic conditions, the utilization of exon 1d seems to be physiologic in the elderly. The specific utilization pattern of multiple exons 1 in elderly breast tissue has not been reported elsewhere. This seems to be because of a difference in subjects as the present study dealt with a number of tissues in the very elderly, who have been free from ovarian function for decades and in whom peripherally localized estrogen-metabolizing enzymes are almost the sole source of estrogens. Among many estrogen-metabolizing enzymes, aromatase plays a key role in estrogen biosynthesis. Estrogen is known to be essential to maintain various physiologic functions. The decrease of serum estrogens has been reported to be related to many geriatric diseases, including osteoporosis [31], Alzheimer disease [32], and atherosclerosis [33]. Although aromatase mRNA levels of EldNorm and ContNorm did not significantly differ, EldNorm exhibited almost twice the levels of aromatase mRNA in comparison with ContNorm. Switching of the preferential use of multiple exons 1 during aging might be advantageous to peripherally produce estrogens by increasing aromatase levels (Figure 1). Given that connective tissue is a major component of normal breast tissue, it cannot be excluded that such phenomena may be observed systemically. Several authors have reported that basal aromatase levels in peripheral stroma are increased in the elderly [34-36]. A systematic study may elucidate the physiologic role of aromatase in the elderly.

Contrary to EldNorm, among which more than 30% of cases were classified as group 'c > d', more than half of cases of EldCa were classified as group 'd > c'. The precise mechanisms of how exon 1d becomes frequently utilized among elderly tissues and how the dominance of exon 1d and exon 1c is converted between EldCa and EldNorm are not known at present. Each exon 1 has its characteristic promoter region [21]. The promoter of exon 1b, a glucocorticoid-stimulated promoter, has a characteristic TATA box-less and GC-box structure, which is often found in the proximal regions of housekeeping genes, and is regulated by class 1 cytokines, tumor necrosis factor-alpha, and glucocorticoids. This may be why transcription from exon 1b does not seem to be so rigid and is widely found in extragonadal tissues, such as skin fibroblasts, adipose tissue, and normal breast tissue. The promoters of exons 1c/1d, in contrast, have not only the rigid structure of CAAT and TATA boxes but also multiple sites of activator protein-1 and cAMP-responsive element (CRE) in proximal regions which enable induction of the effective transcription of the aromatase gene in response to various stimulants, such as prostaglandin E_2 _(PGE_2_), gonadotropins, or growth factors, whose second messenger is cAMP or protein kinase C (PKC) [21]. During senescence, upregulated cyclooxygenase-2 (COX-2) expression and the resulting increase in the production of PGE_2 _in stromal cells, such as fibroblasts or macrophages, have been reported [37-39]. Interaction between carcinoma cells and surrounding stromal cells also elevates COX-2 and PGE_2 _levels [40]. PGE_2 _may be one of the key factors to explain the switching mechanism from exon 1b to exon 1c/1d during aging and carcinogenesis [22,41]. Gonadotropins may be another candidate to explain the mechanism. Markedly low serum estrogen levels in elderly women result in the elevation of gonadotropins, which may lead to switching to exon 1c/1d through cAMP. cAMP will activate PKA, which will lead to the binding of CRE binding (CREB) to CRE in proximal region of exons 1c/1d. Exons 1c/d are typically utilized in the ovary, where aromatase is induced in response to gonadotropins [20]. Our preliminary study suggested that the ovary may alternatively utilize exon 1c or 1d according to the menstrual cycle (unpublished observation). Changes in hormonal circumstances, such as the ratio of gonadotropins (follicle-stimulating hormone/lutenizing hormone) during the menstrual cycle, may be somehow associated with the switching between exon 1c and 1d. In cancerous tissue, there have been many studies to elucidate the DNA-binding proteins, transcription factors, and co-regulators that regulate the alternative utilization of multiple exons 1 [42,43]. For example, S1 (silencer element), which is situated between exons 1c and 1d, negatively regulates aromatase expression in normal breast tissue, suppressing the function of exons 1c/1d in combination with EAR-2/COUP-TF-1/EAR-γ. In cancer tissue, the expression levels of EAR-2/COUP-TF-1/EAR-γ decreased and aromatase expression is upregulated through the binding of ERRα-1 (estrogen-related receptor alpha-1) to S1 [42,43]. Aging and carcinogenesis may somehow affect the constitution of these DNA-binding proteins, transcription factors, and co-regulators. Further study is needed to elucidate the molecular mechanism of switching of the preferential use of multiple exons 1 during aging and carcinogenesis.

Among EldCa, mucinous carcinomas examined were all ER-positive [44,45], were all classified as group 'd > c', and exhibited significantly higher aromatase mRNA levels than other carcinomas. These findings may suggest the importance of peripheral aromatase and estrogens in the pathobiology of mucinous carcinoma, which is a specific histologic type frequently observed in elderly women [24].

## Conclusions

This is the first study to systematically examine the preferential usage of multiple exons 1 of the aromatase gene in breast tissue of the very elderly. Elderly breast tissues typically used exon 1d irrespective of whether the tissues were cancerous or normal. The dominance of exon 1c and exon 1d was converted between cancerous and normal tissues. This seems to at least partly explain the elevation of aromatase mRNA levels in elderly breast cancer. The molecular mechanism of how switching of exons 1 occurs should be further studied.

## Abbreviations

ANOVA: analysis of variance; ContCa: carcinomas from controls; ContNorm: normal tissues from controls; COUP-TF-1: chicken ovalbumin upstream promoter-transcription factor 1; COX-2: cyclooxygenase-2; CRE: cAMP-responsive element; EldCa: carcinomas from the elderly; EldNorm: normal tissues from the elderly; ER: estrogen receptor; Ex 1: multiple exons 1 of the aromatase gene; PCR: polymerase chain reaction; PGE_2_: prostaglandin E_2_; RT: reverse transcription; RT-PCR: reverse transcription-polymerase chain reaction; S1: silencer element.

## Competing interests

The authors declare that they have no competing interests.

## Authors' contributions

NHo designed the study, provided study materials, analyzed data, and drafted the manuscript. KT histologically classified breast carcinomas and scored the immunohistochemical results. MS, TA, FA, and TU provided study materials. GS histologically classified breast carcinomas. NY analyzed mRNA data. NHa designed the study, analyzed mRNA data, and helped to draft the manuscript. All authors read and approved the final manuscript.
